# Preclinical serial shear stress analysis of a novel strut-free fibrillated bioresorbable polymeric endoluminal graft

**DOI:** 10.3389/fcvm.2026.1744904

**Published:** 2026-02-06

**Authors:** Lucas Hatzikostas, Kotaro Miyashita, Rick de Vries, Bart Sanders, Golo von Basum, Kim van Noort, Jouke Dijkstra, Christos V. Bourantas, Tsung-Ying Tsai, Yoshinobu Onuma, Peter Barlis, Patrick W. Serruys, Eric K. W. Poon

**Affiliations:** 1 Department of Medicine, Melbourne Medical School, Faculty of Medicine, Dentistry and Health Sciences, The University of Melbourne, Fitzroy, VIC, Australia; 2 Department of Cardiology, Royal Melbourne Hospital, Parkville, VIC, Australia; 3 Department of Cardiology, University of Galway, Galway, Ireland; 4 STENTiT B.V., Eindhoven, Netherlands; 5 Department of Radiology, Leiden University Medical Center, Leiden, Netherlands; 6 Device and Innovation Centre, William Harvey Research Institute, Queen Mary University of London, London, United Kingdom; 7 Department of Cardiology, Barts Heart Centre, Barts Health NHS Trust, London, United Kingdom; 8 Department of Mechanical Engineering, Faculty of Engineering and Information Technology, The University of Melbourne, Melbourne, VIC, Australia

**Keywords:** bioresorbable vascular scaffold, computational fluid dynamics, endothelial shear stress, optical coherence tomography, resorbable fibrillated scaffold

## Abstract

**Aim:**

To characterise near-wall haemodynamics immediately after implantation of a next-generation, strut-free biorestorative endoluminal graft, evaluate changes over 3 months during resorption, and assess whether early flow patterns may influence subsequent remodelling in two preclinical models.

**Methods:**

Three rabbits and six mini-pigs underwent bilateral implantation of a resorbable fibrillated scaffold (RFS) in peripheral arteries. Intravascular optical coherence tomography (OCT) combined with angiography enabled generation of 29 case-specific three-dimensional reconstructions during a 3-month study period. Pulsatile, non-Newtonian computational fluid dynamics (CFD) quantified the endothelial shear stress (ESS).

**Results:**

OCT demonstrated a continuous endoluminal surface consistent with the strut-free RFS design and revealed a distinct optical transition at the device boundary. Haemodynamic mapping showed low-ESS regions at scaffold edges and broadly unidirectional near-wall flow within the scaffolded segment. Quantitatively, ESS showed a modest, non-significant upward trend during follow-up before stabilising (rabbits: +0.37 Pa, *p* = 0.085; mini-pigs: +0.37 Pa, *p* = 0.091). Higher early ESS correlated with subsequent lumen gain (*ρ*_s_ = 0.50; *p* < 0.001), and serial analyses revealed an evolving association over time.

**Conclusion:**

RFS implantation instated a largely homogeneous ESS profile that evolved with 3-month remodelling, consistent with a dynamic flow-healing interplay that warrants longer-term evaluation through full bioresorption.

## Graphical Abstract

**Figure:**
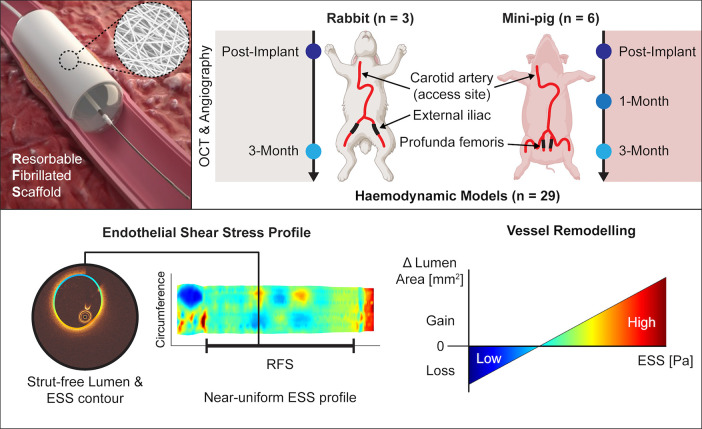
Resorbable fibrillated scaffold (RFS) shear stress assessment in a preclinical study. Bilateral RFS implants in rabbit external iliac and mini-pig profunda femoris arteries were evaluated over 3 months. Endothelial shear stress (ESS) was quantified using three-dimensional geometries reconstructed by OCT-angiographic fusion. The strut-free, continuous-wall scaffold generated a more uniform ESS profile than typical of lattice-based stents, and post-implantation ESS correlated with subsequent lumen remodelling.

## 1 Introduction

Contemporary interventional cardiology relies heavily on drug-eluting stents (DES), which continue to deliver excellent revascularization outcomes across diverse lesions and patient profiles (1). Nevertheless, the permanent metallic lattice can impair vasomotion and mechanotransduction, delay endothelial healing, complicate re-intervention, and precipitate adverse events such as very late stent thrombosis (2). Bioresorbable vascular scaffolds (BVS) were developed to provide temporary support and restore natural vascular function after resorption. While first-generation BVS showed higher early thrombosis and target-lesion failure, these excess risks attenuate with longer follow-up and outcomes approach those of DES (3). Encouraging results in below-the-knee applications have renewed interest in a “cage-free” vessel (4) and driven next-generation designs.

The goal of “biorestorative” scaffolds is to resorb while fostering vascular healing (5). The Resorbable Fibrillated Scaffold (RFS) is one such device which combines a tubular, strut-free design with a porous, fibre-based polymer microarchitecture (STENTiT B.V., Eindhoven, the Netherlands). Preclinical data suggest that its fibrillated (acellular) matrix serves as a template for elastin-rich, organised neotissue ingrowth, and promotes rapid re-endothelialisation (6). Elastin enrichment is noteworthy because it is a hallmark of healthy arterial structure and confers resistance to atherosclerosis (7).

Any intravascular scaffold, including biorestorative devices, alters lumen geometry and reshapes near-wall flow. These geometric changes modify local endothelial shear stress (ESS), a key mechanobiological determinant of endothelial phenotype and vascular health. High-resolution intravascular imaging combined with computational fluid dynamics (CFD) enables case-specific characterisation of microscopic, scaffold-induced flow disturbances, which have been linked to restenosis, thrombosis, and long-term device performance (8–14).

Despite promising preclinical healing features, the haemodynamic performance of the RFS has not been characterised. A first-in-human feasibility study (VITAL-IT 1) is evaluating its use in below-the-knee chronic limb-threatening ischaemia, highlighting the importance of mechanistic data. We therefore combined optical coherence tomography (OCT)-angiography fusion with CFD in two animal models to: (i) map ESS immediately after implantation, (ii) track its 3-month evolution during resorption, and (iii) identify early near-wall haemodynamic patterns that may inform subsequent vascular remodelling.

## 2 Methodology

All procedures were approved by the Utrecht University Institutional Ethical Committee and the Dutch Central Authority for Scientific Procedures on Animals (Rabbits: AVD2290020186144, 04 September 2018; Mini-pigs: AVD22900202216226, 05 June 2023) and conducted at the Gemeenschappelijk Dierenlaboratorium facility, Utrecht. The study complied with ARRIVE guidelines, EU Directive 2010/63, and NIH standards for animal research.

### 2.1 Experimental design

The RFS was fabricated by electrospinning (Vivolta, Waalre, the Netherlands) a poly-lactic-based co-polymer (Corbion Purac, Gorinchem, the Netherlands) into a strut-free tubular construct without anti-proliferative coating. The near-wall haemodynamic performance of the RFS was evaluated in two preclinical models (rabbits and mini-pigs). Both species are established platforms for evaluating the feasibility, safety and biocompatibility of vascular stents (15). As exploratory feasibility studies, group sizes were selected pragmatically rather than by sample size calculations. Blinding was not applied, as all animals received the same intervention and no untreated control group was included.

Three 12–15-week-old male New Zealand White rabbits (Charles River, France; body weight 2.4–2.8 kg) underwent bilateral implantation of 2.0 mm × 10 mm × 220 µm (inner diameter × length × nominal thickness) RFS's in the external iliac arteries via a carotid approach. Angiography and OCT imaging were performed immediately post-implantation and at 3-month follow-up.

The RFS was upscaled to 3.0 mm × 27 mm for implantation in a porcine model to mimic the dimensions of human below-the-knee intervention. Using carotid access, RFS's were implanted bilaterally in the profunda femoris arteries of six female 15–17-month-old Göttingen mini-pigs (Ellegaard, Dalmose, Denmark; body weight 35.0–41.0 kg). Angiography and OCT were performed immediately post-implantation and again at 1- and 3-month follow-ups.

Additional details of animal care, anaesthesia, analgesia, and perioperative management are provided in the Supplementary Methodology 1, 2, with the study design and imaging inclusion/exclusion workflow shown in Supplementary Figure S1.

### 2.2 Image acquisition and analysis

Contrast angiography and intravascular OCT were performed immediately after scaffold implantation and at scheduled follow-up. Angiography was obtained with animals positioned supine in an anteroposterior view for standardized assessment of vessel patency and scaffold placement. Cine runs were recorded at 15 frames/s and exported in DICOM (1,024 × 1,024 pixels) format. OCT imaging (St. Jude Medical, St. Paul, MN, USA) used an automatic pullback of 18 mm/s at 180 frames/s, following intracoronary contrast flushing to clear the imaging field.

Imaging data was analysed offline by an independent core laboratory (CORRIB Laboratory, University of Galway, Ireland). For each case, two anatomical segments were defined:

1. Region of interest (ROI): the vessel portion reconstructed into a three-dimensional model for haemodynamic analysis
2. Scaffolded Segment (RFS): the vessel length treated with the RFS, delimited by its proximal and distal edges.

Side-branch landmarks visible in both angiography and OCT were catalogued by their axial position and circumferential orientation. These fiduciaries enabled co-registration of the two modalities and ensured that serial analyses surveyed the same vessel segment at each follow-up. ROI boundaries were set at the nearest proximal and distal landmarks; if a landmark lay within 3 mm of a scaffold edge, the next closest landmark was selected instead. Under OCT, the side branch location was defined at the carina, corresponding to the frame immediately before confluence of the side-branch and main vessel.

**Figure 1:**
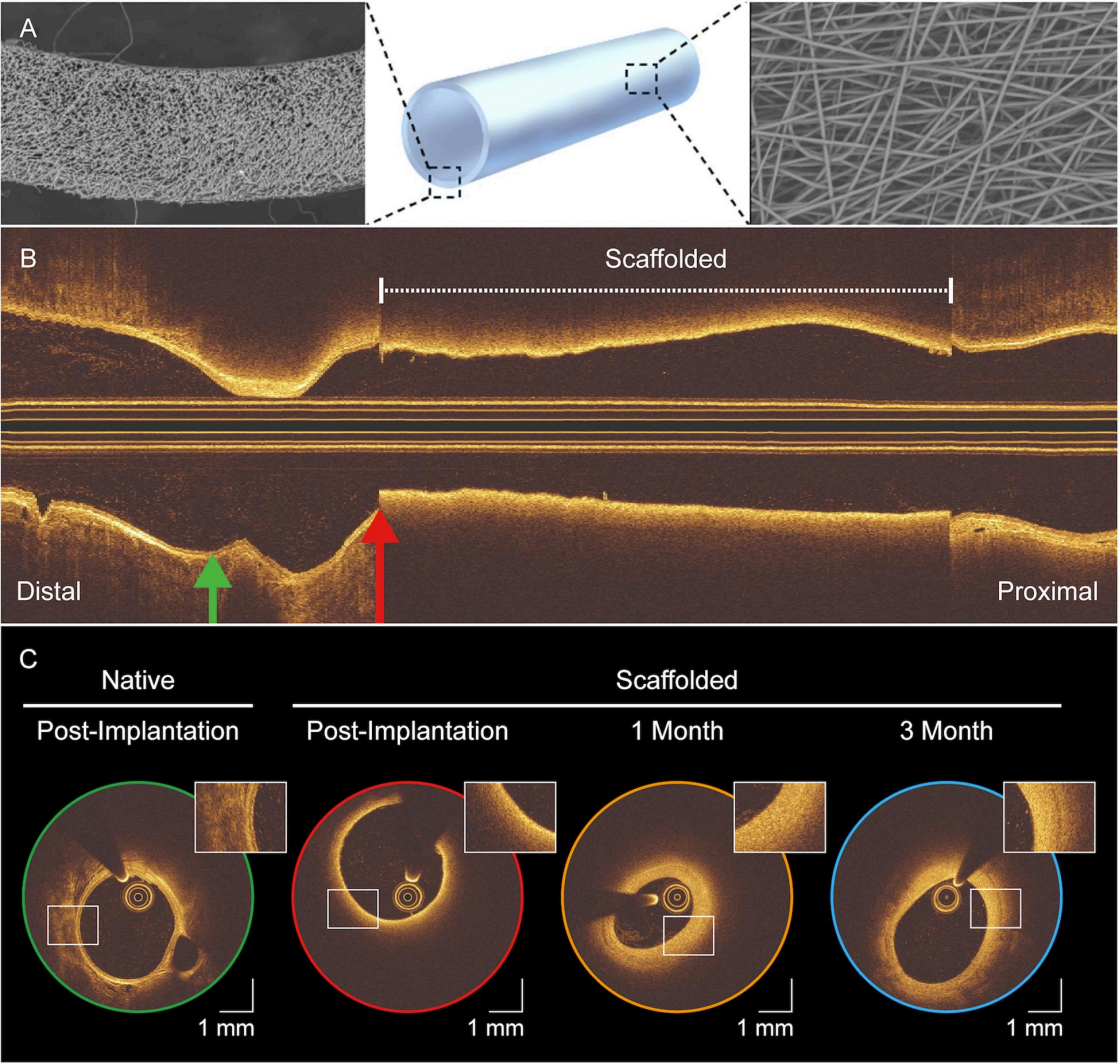
Serial intravascular OCT of the resorbable fibrillated scaffold. **(A)** 3D rendering of the scaffold's tubular, strut-free design with scanning electron microscopy insets of the cross-section (left) and outer surface (right). **(B)** Post-implantation longitudinal OCT view; dashed bracket marks the scaffolded segment. Green and red arrows indicate sites of the cross-sections in **(C)**, which shows axial OCT images at post-implantation (green, red), 1-month (yellow), and 3-month (blue), with magnified insets highlighting scaffold-vessel interaction over time. Scale bar: 1 mm. Compared with the native vessel (green), reduced backscatter clearly delineates the device margins (red), producing a characteristic “blurring” that diminishes with time.

Unlike other bioresorbable scaffolds which incorporate metallic markers or radio-opaque composites (16), the RFS lacked such features, necessitating OCT for edge localization (see Figure 1). Nevertheless, the RFS exhibited a distinctive optical signature: its fibrillated architecture produces near-field optical scatter that shadows underlying anatomic structures, creating an abrupt transition. Scaffold edges were identified in the OCT frame where this optical scatter effect completely encircled the lumen (see Supplementary Video S1).

OCT pullbacks were analysed at 100 µm intervals along both the ROI and scaffold-treated (RFS) segments. Three metrics were calculated: (i) segment length (mm); (ii) minimum lumen area (MLA, mm^2^); and (iii) lumen volume (LV, mm^3^) (17).

### 2.3 Three-dimensional reconstruction

Three-dimensional reconstructions of the case-specific arterial geometry were performed by fusing the monoplane-derived angiographic centreline with OCT lumen cross-sections. Typically, in the coronary arteries two angiographic views separated by at least 25 degrees are preferred to ensure an accurate 3D centreline reconstruction. However, in this case, the vessel centreline was extracted from the only available single anteroposterior (AP) angiogram. As both external iliac and profunda femoris arteries lie predominantly in the coronal plane, the AP projection aligns this plane with the detector, minimizing foreshortening and rendering a monoplane acquisition sufficient for 3D reconstruction.

Vessel lumen contours were then semi-automatically extracted from OCT data within the ROI (QCU-CMS-v4.69, Leiden University Medical Centre, Leiden, the Netherlands). Finally, according to expert guidelines (18), these contours were precisely placed perpendicularly to the angiographic centreline using the catalogued anatomic landmarks to estimate their absolute orientation (MATLAB R2024b, MathWorks Inc., Natick, MA, United States) (19). Unlike conventional strut-based stent designs, the RFS forms a closed, tubular conduit without lateral openings. As a result, blood flow can enter and exit the graft only at its proximal and distal margins, and the grafted segment was modelled as a single conduit consistent with its physical structure. The pipeline for co-registration and three-dimensional reconstruction is shown in Supplementary Figure S2, and an overview of the final reconstructed vessel geometries is provided in Supplementary Figure S3.

### 2.4 Haemodynamic assessment

Computational fluid dynamics (CFD) was employed to evaluate the haemodynamic environment of the RFS. For each reconstructed case, a computational mesh was generated for accurate haemodynamic modelling. This finite-volume mesh consisted of at least five layers of prism elements with the initial prism layer thickness set to 0.05 mm and a 10% increment with each subsequent layer. This layering strategy ensured adequate resolution near the lumen surface to accurately capture the velocity gradient. To ensure spatial independence, mesh resolution was selected using physics-based near-wall criteria appropriate for laminar arterial flow, targeting y^+^ < 1 based on the mean blood flow velocity and proximal (inlet) diameter (20). The resulting smallest mesh elements were then used to determine the temporal resolution from the local convective time scale, with time-step selection primarily constrained by flow pulsatility to ensure smooth representation of systolic acceleration and deceleration. Further details are provided in Supplementary Methodology 3.

CFD modelling was performed using an in-house validated finite-volume method based on the robust open-source CFD toolbox (OpenFOAM v7, The OpenFOAM Foundation Ltd., London, UK). Appropriate boundary conditions were selected for each case, with mean inflow velocity estimated using the Thrombolysis in Myocardial Infarction (TIMI) frame count for the rabbit models (21). In mini-pigs, several angiograms were acquired only after full contrast opacification of the vessel, precluding reliable TIMI-based estimates; therefore, an empirically derived flow-diameter scaling law was applied in these cases (22). Given the lack of direct blood velocity measurements, a generic peripheral arterial waveform was prescribed at the inlet for both animal cohorts to represent physiological pulsatility, with the mean inflow magnitude scaled to match the aforementioned species-specific velocity estimates (23, 24). As blood viscosity was not measured directly, we adopted the standard Quemada constitutive model (25), consistent with prior studies showing that the animal and human blood share similar shear-thinning behaviour (26, 27). In the absence of electrocardiogram data, heart rates of 150 beats/minute (rabbits) (28), and 75 beats/minute (mini-pigs) (29) were assumed. Three cardiac cycles were simulated to dissipate initial transients. Relevant haemodynamic metrics were collected throughout the third cardiac cycle and were time-averaged. Key CFD set-up conditions are summarized in Supplementary Table S1.

We evaluated several haemodynamic indices to characterise near-wall flow. The primary measure was endothelial shear stress (ESS) (see Supplementary Video S2). Additional indices included the ESS gradient (ESSG), transverse ESS (transESS), oscillatory shear stress (OSI), and the relative residence time (RRT), each capturing complementary aspects of shear stress magnitude, direction, and flow stagnation (see Supplementary Table S2). Results were projected onto a two-dimensional “carpet view” by virtually opening the vessel along its longitudinal direction and unrolling the luminal surface into a planar map spanning axial length and circumferential position. This post-processing representation enables continuous visualisation of near-wall haemodynamic patterns along the vessel at a uniform resolution (0.1 mm × 1°).

### 2.5 Statistical analysis

Continuous variables were summarised as mean ± SD when approximately normally distributed, or as median (IQR) when skewed. Categorical variables were expressed as counts (%). Temporal changes in OCT- and CFD-derived parameters were analysed using linear mixed-effects models, which account for within-subject correlation and accommodate unbalanced longitudinal data. Time [post-implantation (PI), 1 month (1M), 3 months (3M)] was specified as a fixed effect, and Animal ID was included as a random intercept. When two or more time-point contrasts were available (e.g., in mini-pigs: PI, 1M, 3M), all pairwise comparisons were Bonferroni-adjusted. For rabbits (two time points: PI and 3M), only a single contrast was evaluated without adjustment. Model-estimated effects are reported as mean differences (Δ) with corresponding 95% confidence intervals (CI) and adjusted *p*-values. Associations between ESS and temporal change in lumen cross-sectional area (ΔCSA) were examined using Spearman's rank correlation (*ρ*_s_). A two-tailed *p* < 0.05 was considered statistically significant. All analyses were conducted in R (version 4.5.0; R Foundation for Statistical Computing, Vienna, Austria) using the *lme4*, *lmerTest*, and *emmeans* packages.

## 3 Results

### 3.1 Intravascular OCT imaging features

Twenty-nine OCT pullbacks were analysed (8 rabbits, 21 mini-pigs), comprising 15 504 frames. Key vessel metrics were evaluated using linear mixed-effects modelling, with model-derived temporal contrasts provided in Supplementary Table S3 for full inferential detail. Case-specific scaffold lengths are illustrated in Supplementary Figure S4.

In rabbits, scaffold length shortened by −1.55 mm [95% CI (−1.87, −1.23); *p* < 0.001] over 3 months, while changes in lumen volume and minimal lumen area within the scaffolded segment were not significant. In mini-pigs, marked early reductions occurred by 1 month: scaffold length −4.64 mm [95% CI (−6.19, −3.10); *p* < 0.001], MLA −3.27 mm^2^ [95% CI (−4.70, −1.84); *p* < 0.001], and lumen volume −97.86 mm^3^ [95% CI (−130.50, −65.21); *p* < 0.001]. Between 1 and 3 months, dimensions remained stable (all *p* > 0.99). Across the reconstructed vessel, similar trends were observed.

### 3.2 Qualitative assessment of shear stress post-implantation

**Figure 2:**
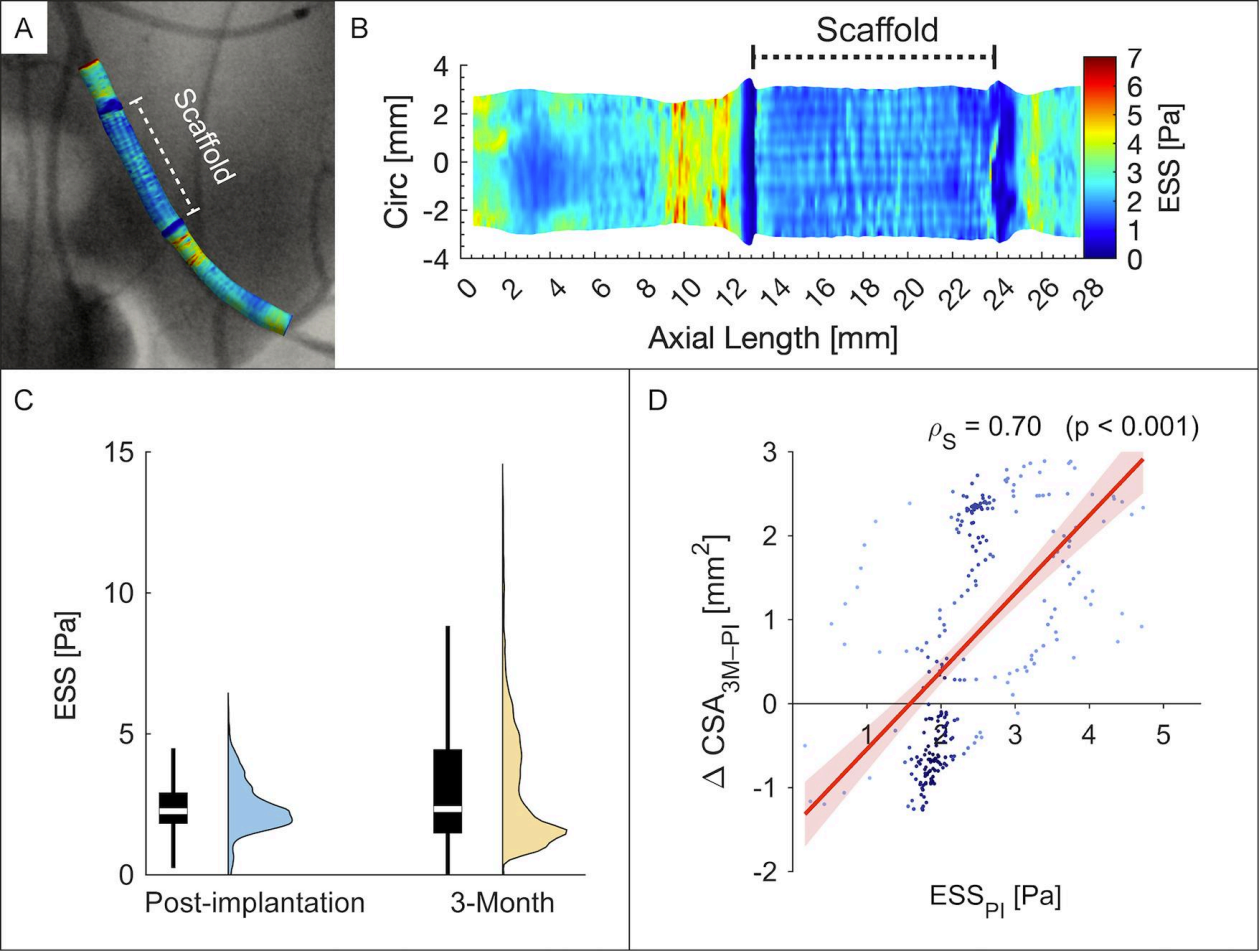
Endothelial shear stress in a representative rabbit external iliac artery. **(A)** Three-dimensional reconstruction of the left iliac artery showing post-implantation ESS co-registered with angiography. **(B)** Carpet plot of ESS distribution along vessel length and circumference; dashed bracket marks the scaffolded segment. **(C)** Violin/boxplots of ESS at post-implantation (blue) and 3 months (yellow). **(D)** Scatterplot of post-implantation ESS (ESS_PI_) versus 3-month change in lumen cross-sectional area [ΔCSA_3M–PI_ (mm^2^)]; points coloured by density (dark to light blue). Circ, circumference.

Post-implantation, both species revealed characteristic shear stress patterns. Clearly defined regions of low ESS were observed at the scaffold edges (Figures 2A,B). Within the scaffold, ESS was broadly homogeneous; rabbit cases additionally exhibited a subtle criss-cross motif. Within the RFS-segment, multidirectional indices demonstrated predominately unidirectional near-wall flow patterns (Supplementary Figure S5).

### 3.3 Temporal changes in shear stress

**Figure 3:**
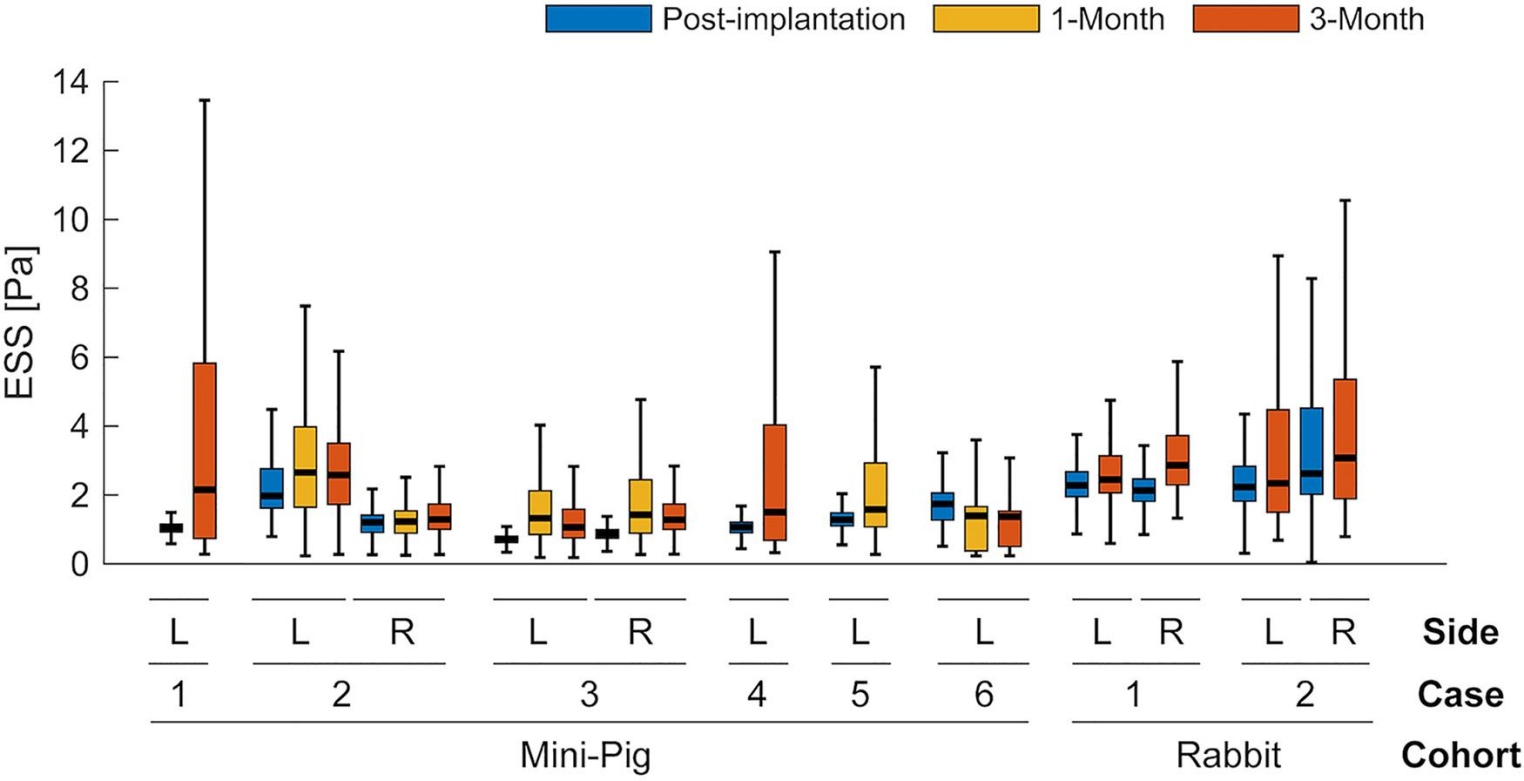
Temporal changes in endothelial shear stress (ESS) after scaffold implantation. Box-and-whisker plots show ESS at post-implantation (blue), 1-month (yellow), and 3-month (red) for each case and vessel side (L, left; R, right). Boxes indicate median and IQR; whiskers extend to 1.5 × IQR. Complementary plots of multidirectional metrics are shown in Supplementary Figure S5.

ESS showed a non-significant upward tendency in both species (see Figure 3). In rabbits, ESS increased by +0.37 Pa [95% CI (−0.09, 0.83); *p* = 0.085, single contrast]. In mini-pigs, ESS rose by a similar +0.37 Pa at 1 month vs. baseline [95% CI (−0.05, 0.80); *p* = 0.091] and then plateaued through 3 months. Overall, changes did not reach statistical significance after adjustment, but both cohorts exhibited the same directional trend. Complementary indices (ESSG, transESS, OSI, RRT) exhibited comparable temporal fluctuations (see Supplementary Figure S6).

### 3.4 Shear stress and luminal remodelling

**Figure 4:**
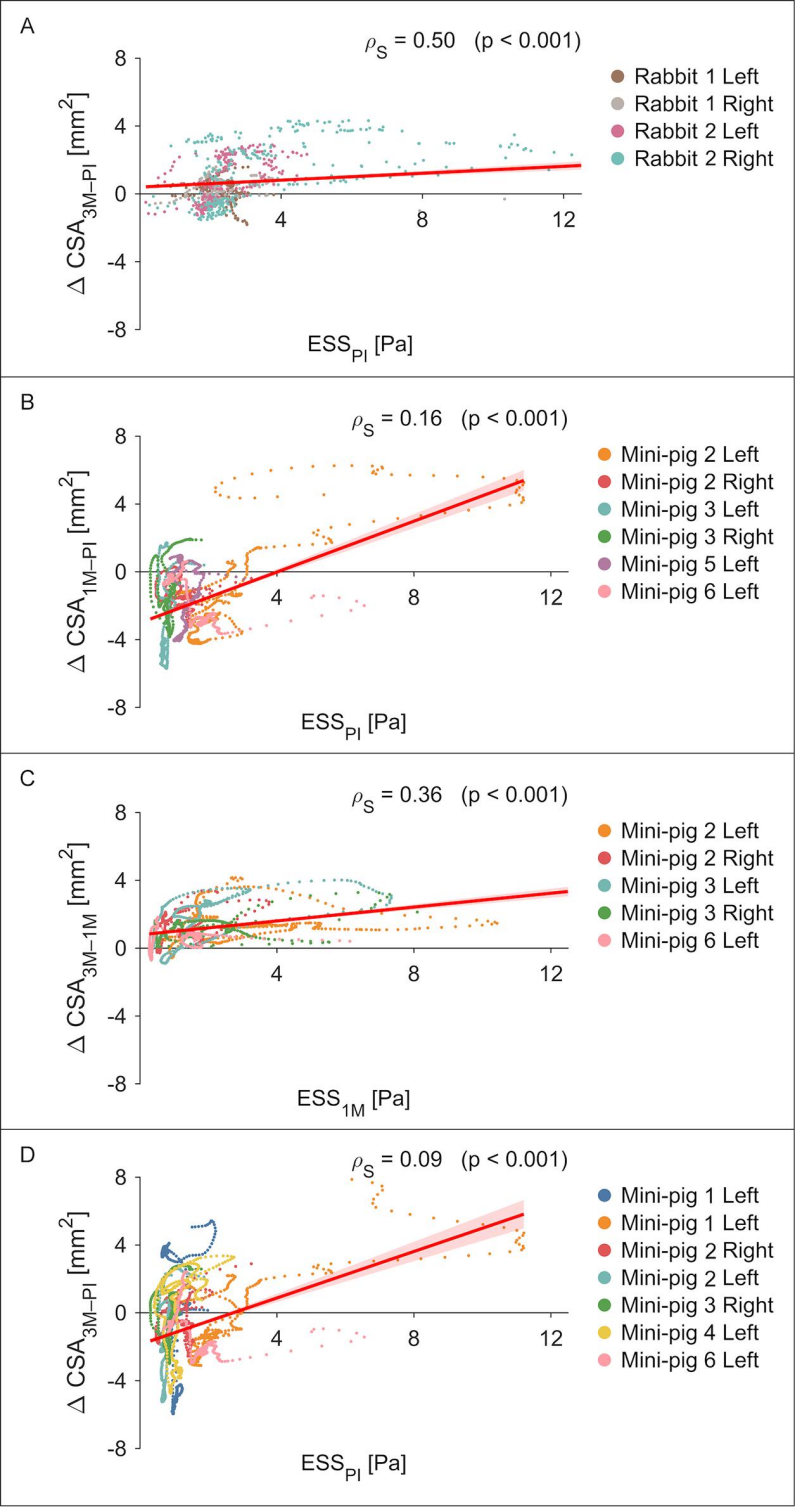
Endothelial shear stress and subsequent changes in lumen cross-sectional area. Scatterplots are shown for each case (unique colours): rabbits at **(A)** post-implantation vs. 3-month, and mini-pigs at **(B)** post-implantation vs. 1-month, **(C)** 1-month vs. 3-month, and **(D)** post-implantation vs. 3-month. For each cross-section, ESS was averaged over the full circumference. Spearman's rank correlation (*ρ_s_*) and *p*-value are reported in each panel. Linear regression lines with 95% confidence intervals (red shading) illustrate the trend.

Figure 4 presents the association between ESS and ΔCSA at each time interval. In rabbits, a moderate positive correlation was observed between post-implantation ESS and ΔCSA at 3 months (*ρ_s_* = 0.50, *p* < 0.001, *n* = 4; Figure 4A), with one vessel showing a notably stronger relationship (*ρ_s_* = 0.70, *p* < 0.001; Figure 2D). In mini-pigs, post-implantation ESS correlated weakly with ΔCSA at 1 month (*ρ_s_* = 0.16, *p* < 0.001, *n* = 6; Figure 4B). However, a strengthened correlation emerged between ESS at 1 month and ΔCSA at 3 months (*ρ_s_* = 0.36, *p* < 0.001, *n* = 5; Figure 4C).

## 4 Discussion

In this study, the near-wall haemodynamic performance of the RFS was successfully evaluated for the first time in two preclinical models. The scaffolded segment displayed a distinctive shear profile at post-implantation, marked by minimal spatial variability in ESS (see Figures 2A,B). This homogeneity likely reflects the scaffold's strut-free design, supported by OCT findings that revealed a continuous endoluminal surface (see Figure 1C). However, rabbit haemodynamic maps exhibited a faint criss-cross ESS motif. In such cases, OCT revealed subtly polygonal, approximately decagonal luminal cross-sections rather than circular ones (see Supplementary Video S1), plausibly reflecting mild oversizing or nuances in balloon-scaffold mounting. The motif was discernible only with ultra-high-resolution OCT, underscoring its value for resolving fine, local flow phenomena (10, 12).

Protruding stent struts create microscopic obstacles for flowing blood. This irregular geometry generates flow separation, recirculation, and low and oscillatory shear niches, conditions linked to inflammation, thrombosis and neointimal hyperplasia (30–35). ABSORB BVS shows an initial high–low ESS pattern across successive struts that homogenises by five years as the scaffold resorbs (9). In contrast, the RFS installs a smooth, strut-free endoluminal contour that mimics the native vessel (“vasculo-mimetic”). Consistent with this design, near-wall haemodynamics was predominantly unidirectional, lacking zones of low or oscillatory ESS (see Supplementary Figure S5).

We sought to determine whether this favourable haemodynamic environment translated into reduced neointimal formation. However, OCT-based evaluation was hindered by optical backscatter from the fibrillated scaffold microarchitecture, which obscured delineation of tissue-scaffold borders and prevented precise quantification (see Figure 1C). Consequently, although the ESS distributions suggests a favourable haemodynamic environment free of disturbed near-wall flow, which may mitigate maladaptive intimal thickening, direct confirmation requires histological validation and complementary imaging modalities (36). Nevertheless, the identification of this unique, device-specific optical artefact represents an important observation in OCT characterisation of the RFS.

ESS correlated with lumen area changes, with a stronger association in rabbits than in mini-pigs. This difference may partly reflect inflow boundary condition assumptions, using case-specific TIMI frame-counts in rabbits vs. a generalized flow-diameter scaling law in mini-pigs. Nevertheless, unlike conventional open- or closed-cell lattice stents that expose the wall to shear stress, the RFS's continuous surface tubular design partially shields the vessel immediately post-implantation. However, rapid re-endothelialisation observed preclinically (37) likely reconstitutes the shear-sensing interface soon after. In mini-pigs, ESS at the first follow-up correlated more strongly with subsequent lumen changes than post-implant ESS (see Figure 4). Although causality remains unproven, this shift may reflect evolving endothelial responsiveness to ESS and suggests that tissue integration into the fibrillated scaffold, as observed preclinically (6), could promote vascular restoration.

Current regulatory evaluations of stents, such as those by the FDA, focus largely on mechanical performance and do not account for haemodynamic behaviour. Future scaffold designs should incorporate CFD-informed assessments (38). At the proximal and distal scaffold edges, OCT revealed distinct “step-up” and “step-down” transitions, respectively. These abrupt shifts between scaffolded and native vessel segments induced recirculating, low-velocity flow, forming low-shear stress rings. Such edge effects are clinically relevant, as they can promote restenosis and thrombosis (30, 39). Despite its strut-free profile, edge geometry remained a key determinant of local flow. Owing to modest scaffold shortening, assessment of shear-induced remodelling at device edges was limited. Nevertheless, lumen loss at follow-up consistently localised to edge regions that had been exposed to low ESS at implantation (see Supplementary Figure S5). Building on prior findings (13, 38, 40), thin, tapered, or curved edge transitions can reduce flow separation, increase local ESS, and minimize recirculation. However, these haemodynamic benefits must be balanced against the reduced mechanical strength of polymer-based materials compared to metals, as thinner walls risk radial collapse. Tapered edges provide validated haemodynamic advantages but must meet structural requirements for acute support and long-term safety. Balancing mechanical stability with optimal shear characteristics is critical for promoting vascular healing.

This preliminary animal study has several limitations. This preliminary animal study has several limitations. First, the sample size was modest, and findings should be considered exploratory. However, each animal underwent bilateral implantation with serial follow-up, yielding repeated haemodynamic and morphological measurements per subject, providing a rich dataset appropriate for early-stage feasibility and hypothesis-generating analyses. Second, imaging was monoplane rather than biplane; however, vessels anatomy was largely planar, limiting foreshortening. Finally, rabbit inflows estimates were case-specific (TIMI), whereas mini-pig simulations relied on scaled estimates.

RFS implantation instated a broadly homogeneous ESS profile, consistent with its strut-free, continuous-wall design. This pattern evolved with vessel remodelling, underscoring the dynamic interplay between shear stress and vascular healing. These preliminary findings warrant longer-term evaluation to elucidate scaffold-flow interactions throughout full bioresorption and to enable comparison with lattice-based stents.

## Data Availability

The original contributions presented in the study are included in the article/supplementary material, further inquiries can be directed to the corresponding authors.
