# Supplementary material for: Preclinical serial shear stress analysis of a novel strut-free fibrillated bioresorbable polymeric endoluminal graft
Source: Front Cardiovasc Med. 2026 Feb 6;13:1744904. doi: 10.3389/fcvm.2026.1744904 (PMC12920521; doi:10.3389/fcvm.2026.1744904)
Supplement: Supplementary file 1 [file Datasheet1.docx]

Supplementary Material

# Supplementary Methodology

## Supplemental Methodology 1. Animal Care

Rabbits were housed individually in straw-bedded cages with olfactory access to neighbouring cages to minimise aggression. Environmental enrichment included chew items, shelters, and tunnels. Mini-pigs were group-housed in straw-bedded pens, with up to two companions per pen. Enrichment consisted of hard plastic balls and chained hard-plastic or wooden toys. All animals were maintained at room temperature on a 12-hour light/dark cycle with ad libitum access to standard chow and water. Welfare was monitored throughout and included assessment of body weight, skin condition, hydration status, wound infection, pain indicators, lameness, and distal necrosis relative to the implantation site. At study completion, all animals were euthanised by intravenous administration of an overdose of pentobarbital.

## Supplemental Methodology 2. Anaesthesia, Analgesia, and Perioperative Care

Animal anaesthesia and positioning protocols were consistently maintained across imaging sessions. In rabbits, premedication comprised subcutaneous acepromazine (1.5 mg/kg), methadone (1 mg/kg) and midazolam (4.2 mg/kg), with anaesthetic induction by intravenously midazolam (0.57 mg/kg) and sufentanil (0.0037 mg/kg). Maintenance infusion rates were midazolam 5.7 mg/kg/h and sufentanil 0.037 mg/kg/h. Analgesia consisted of subcutaneous meloxicam (0.8 mg/kg) administered pre-operatively and on post-operative day 1. Anaesthetic depth was monitored by assessing heart rate, blood pressure, capnography, body temperature, and the absence of eyelid and pedal-withdrawal reflexes. Antithrombotic prophylaxis, clopidogrel (10 mg/100 mL) and acetylsalicylic acid (10 mg/100 mL), was delivered via drinking water throughout the entire study; intravenous heparin (500 IU) was repeated every 90 minutes as required.

In mini-pigs, animals were premedicated with intramuscular ketamine (13 mg/kg), midazolam (0.7 mg/kg) and atropine (0.05 mg/kg), induced with intravenous propofol (3 mg/kg), and maintained on propofol 4.5 mg/kg/h and remifentanil 0.03 mg/kg/h infusions. Analgesia included a 20 mg transdermal buprenorphine patch (35 µg/h release over 4 days), intraprocedural meloxicam (0.4 mg/kg IV), and meloxicam (0.4 mg/kg IM) once daily for the first two days after surgery. Depth of anaesthesia was assessed by monitoring heart rate, blood pressure, ECG, capnography, pulse oximetry, body temperature, and the absence of eyelid reflexes.

Mini-pigs received an antithrombotic regimen of oral clopidogrel: 300 mg one day prior to and on the day of scaffold implantation, then 150 mg daily for the remainder of the study. Acetylsalicylic acid was administered orally at 80 mg/day throughout. During the first procedure, one animal was given an IV bolus of 5 000 IU heparin, which caused a hematoma at the carotid access site. Thereafter, the intraoperative heparin dose was revised to 100 IU/kg body weight, with additional 100 IU/kg doses every 90 minutes as required.

## Supplemental Methodology 3. Computational Fluid Dynamics Spatial and Temporal Resolution Assessment

Spatial Resolution

Based on the bulk (cross-sectionally averaged) inlet velocity and inlet diameter, flow remained laminar across all simulations, with inlet Reynolds numbers in the range of ${Re}_{inlet}\approx39-80 \left( \mathrm{mean}\approx53 \right).$

To assess spatial resolution conservatively, all mesh-adequacy estimates were evaluated under worst-case conditions at the minimum lumen area (MLA), where velocities and near-wall shear gradients are expected to be maximal.

For laminar internal flow, a characteristic viscous length scale can be estimated as $l_{v}\approx D/\sqrt{Re}$, where $D$ is the characteristic diameter and $Re$ the Reynolds number.

Using MLA-based quantities, $D_{MLA}=1.31-3.07\mathrm{mm} \left( \mathrm{mean}\approx2.0 \mathrm{mm} \right),$and ${Re}_{MLA}\approx42-165 \left( \mathrm{mean}\approx83 \right),$the resulting viscous length scale was $l_{v, MLA}\approx107-457 \mu m \left( \mathrm{mean}\approx240 \mu m \right).$

Near-wall resolution was additionally assessed using the criterion $y^{+}\leq1$ (1), with $y^{+}=({u_{\tau}y)}/v$, where $y$ is the wall-normal distance to the centre of the first computational cell, $v$ is the kinematic viscosity, and $u_{\tau}$is the frictional velocity. Although $y^{+}$ originates from wall-scaling arguments developed in turbulent flows, here it is used purely as a non-dimensional measure of near-wall resolution, linking first-cell height to viscous length scales and wall shear gradients. The frictional velocity was estimated from the bulk velocity using $u_{\tau}=U_{bulk}\sqrt{C_{f}/2}$, where $C_{f}\approx16/Re$, with Reynolds number defined locally at the MLA. The MLA-based bulk velocity was obtained from incompressible continuity, $U_{bulk, MLA}=Q/A_{MLA}=U_{bulk, inlet}\times{(A}_{inlet}/A_{MLA}),$giving $U_{bulk, MLA}= 0.06-0.48 m/s \left( \mathrm{mean}\approx0.17 m/s \right).$

Enforcing $y^{+}$=1 yields a maximum allowable first-cell wall distance of $y_{y^{+}=1}=v/u_{\tau, MLA}\approx38-162 \mu m \left( \mathrm{mean}\approx85 \mu m \right).$

The smallest near-wall element size used in the simulation was $\Delta x_{wall}=50 \mu m.$

Under the worst-case MLA conditions:

1. $\Delta x_{wall}\leq l_{v, MLA}$ in all cases, indicating adequate resolution of the viscous shear layer.
2. $\Delta x_{wall}\leq y_{y^{+}=1}$ in most cases, with marginal exceedance of the $y^{+}\leq1$ criterion in two cases only, reflecting the conservative nature of MLA-based assessment.

For the majority of simulations, the near-wall spacing therefore provided a safety factor of approximately $y_{y^{+}=1}/\Delta x_{wall}\approx2-3,$ accounting for the increase in bulk velocity and corresponding thinning of viscous length scales associated with lumen narrowing at the MLA.

Temporal Resolution

Temporal resolution was selected to resolve the imposed pulsatile inflow waveform and the associated global convective transport. For the minipig simulations, a timestep of $\Delta t=1.25 \mathrm{ms}$ was used with a cardiac cycle duration of $CC=0.8 s$, while for rabbit simulations $\Delta t=0.625 \mathrm{ms}$ was used with $CC=0.4 s$. In both species, this corresponds to approximately 640 timesteps per cardiac cycle, which therefore resolves the rapid acceleration and deceleration phases of systole where temporal gradients are maximal.

A global convective time scale was estimated as $T_{conv,global}\approx D/U,$ using characteristic lumen diameter and bulk velocity scales. In minipigs, $T_{conv,global}$ranged from approximately 4.6 to 52.5 ms (mean ≈ 19.5 ms), while in rabbits it ranged from approximately 2.9 to 14.9 ms (mean ≈ 8.3 ms), such that $\Delta t$ was much smaller than $T_{conv,global}$ in all cases.

As a conservative worst-case check, a local convective scale based on the smallest near-wall spacing was also evaluated, $T_{conv,local}\approx\Delta x/u$, using $\Delta x=50 \mu m$ and MLA-based bulk velocities as an upper-bound estimate. Under this worst-case condition, $T_{conv,local}$ ranged from 0.16 to 0.87 ms in minipigs (mean ≈ 0.41 ms) and from 0.11 to 0.38 ms in rabbits (mean ≈ 0.25 ms).

Last but not least, we further confirmed numerical stability by cross checking with Courant-Friedrichs-Lewy (CFL) numbers, where CFL was estimated as $(U\Delta t)/\Delta x$, using the smallest spatial discretisation ($\Delta x=50 \mu m$). CFL was evaluated using both inlet bulk velocity (representative conditions) and MLA-based bulk velocity (conservative upper bound). In minipigs, ${CFL}_{inlet}$ ranged from 1.36 to 1.71 (mean ≈ 1.47), while ${CFL}_{MLA}$ ranged from 1.44 to 7.86 (mean ≈ 3.77). In rabbits, ${CFL}_{inlet}$ ranged from 1.05 to 1.73 (mean ≈ 1.28), while ${CFL}_{MLA}$ ranged from 1.63 to 5.95 (mean ≈ 2.95). As an implicit solver was employed, CFL values are reported solely as indicators of stable time integration rather than as criteria for temporal accuracy.

# Supplementary Tables

**Supplementary Table 1.** Heart rate is expressed in beats per minute (BPM); inlet velocity in centimetres per second (cm/s). “CPUs” denotes the number of processing cores per simulation. Mesh resolution is reported as the total number of finite-element cells (mean ± standard deviation).

| Parameter | Rabbit | Mini-pig | Total |
| --- | --- | --- | --- |
| CFD Simulations [n] | 8 | 21 | 29 |
| Heart Rate [BPM] | 150 | 75 | – |
| Inlet Velocity [cm/s] | 10.23 ± 1.81 | 5.91 ± 0.40 | – |
| CPUs | – | – | 48 |
| Mesh Elements [n] | – | – | 889,568 ± 301,478 |

**Supplementary Table 2.** OCT-derived Near-wall Hemodynamic Metrics.

| Metric & Units | Description |
| --- | --- |
| Time-Averaged Endothelial Shear Stress (ESS) [Pa] | Shear stress magnitude averaged over the cardiac cycle. |
| ESS Gradient (ESSG) [Pa/mm] | Spatial variation of shear stress along the vessel wall. |
| Transverse ESS (transESS) [Pa] | Shear stress magnitude perpendicular to the primary flow. |
| Oscillatory Shear Index (OSI) [Dimensionless] | Degree of local flow oscillation, backward and forward. |
| Relative Residence Time (RRT) [1/Pa] | Reflects the duration of time that blood spends near the wall, considering the combined effects of low ESS and oscillatory flow patterns |

**Supplementary Table 3.** Model-based temporal contrasts of intravascular OCT-derived vessel metrics. Quantitative intravascular OCT parameters are expressed as model-estimated temporal changes (Δ) with 95% confidence intervals (CI) and p-values derived from linear mixed-effects models, which account for within-animal correlation and unbalanced longitudinal sampling. Reported metrics include length (mm), minimal lumen area (MLA, mm²), and lumen volume (LV, mm³), each evaluated for the full reconstructed vessel (region of interest, ROI) and the scaffolded segment (resorbable fibrillated scaffold, RFS). In rabbits, only post-implantation (PI) versus 3-month (3M) contrasts were assessed, whereas in mini-pigs, PI, 1-month (1M), and 3M time points were compared using Bonferroni-adjusted post-hoc contrasts to control for multiple testing.

| Rabbits | Contrast | | Δ | 95% CI | p-value |  |
| --- | --- | --- | --- | --- | --- | --- |
| Length _ROI_ [mm] | | 3M versus PI | 1.77 | | [+0.56, +2.99] | 0.019 |
| MLA _ROI_ [mm^2^] | | 3M versus PI | -0.19 | | [-0.99, +0.60] | 0.495 |
| LV _ROI_ [mm^3^] | | 3M versus PI | 25.21 | | [-4.87, +55.29] | 0.076 |
|  | |  |  | |  |  |
| Length _RFS_ [mm] | | 3M versus PI | -1.55 | | [-1.87, -1.23] | <0.001 |
| MLA _RFS_ [mm^2^] | | 3M versus PI | -0.51 | | [-1.40, +0.38] | 0.166 |
| LV _RFS_ [mm^3^] | | 3M versus PI | -6.55 | | [-15.91, +2.81] | 0.112 |
| Mini-pigs | Contrast | | Δ | 95% CI | p-value |  |
| Length _ROI_ [mm] | | 3M versus PI | 0.64 | | [-0.40, +1.67] | 0.334 |
|  | | 1M versus PI | -1.07 | | [-2.17, +0.03] | 0.057 |
|  | | 3M versus 1M | 1.71 | | [+0.55, +2.87] | 0.005 |
| MLA _ROI_ [mm^2^] | | 3M versus PI | -1.89 | | [-3.62, -0.16] | 0.032 |
|  | | 1M versus PI | -2.33 | | [-4.13, -0.53] | 0.011 |
|  | | 3M versus 1M | 0.44 | | [-1.41, +2.30] | >0.99 |
| LV _ROI_ [mm^3^] | | 3M versus PI | -26.76 | | [-51.41, -2.12] | 0.032 |
|  | | 1M versus PI | -77.7 | | [-103.83, -51.57] | <0.001 |
|  | | 3M versus 1M | 50.94 | | [+23.46, +78.42] | <0.001 |
|  | |  |  | |  |  |
| Length _RFS_ [mm] | | 3M versus PI | -5.12 | | [-6.60, -3.65] | <0.001 |
|  | | 1M versus PI | -4.64 | | [-6.19, -3.10] | <0.001 |
|  | | 3M versus 1M | -0.48 | | [-2.08, +1.12] | >0.99 |
| MLA _RFS_ [mm^2^] | | 3M versus PI | -2.83 | | [-4.20, -1.46] | <0.001 |
|  | | 1M versus PI | -3.27 | | [-4.70, -1.84] | <0.001 |
|  | | 3M versus 1M | 0.44 | | [-1.03, +1.91] | >0.99 |
| LV _RFS_ [mm^3^] | | 3M versus PI | -89.33 | | [-120.52, -58.13] | <0.001 |
|  | | 1M versus PI | -97.86 | | [-130.50, -65.21] | <0.001 |
|  | | 3M versus 1M | 8.53 | | [-25.22, +42.28] | >0.99 |

# Supplementary Figures

**Supplementary Figure 1.** Study design and dataset selection process. Exclusion of acquired data reflects strict co-registration and image quality criteria. Evaluable and therefore included data is broken down by species and time point.

**Supplementary Figure 2.** Three-dimensional reconstruction and CFD Pipeline. **(A)** Fusion of monoplane coronary angiogram with intravascular OCT images. Co-registration of the X-ray angiogram and axial OCT frames, each OCT slice positioned perpendicular to the angiographic centerline. Colored halos indicate key anatomical landmarks and scaffold margins (red = distal landmark; green = distal scaffold edge; blue = proximal scaffold edge; purple = proximal landmark). **(B)** Resulting 3D vessel model overlaid on the angiogram, shown with a representative pulsatile peripheral-artery waveform applied as an inlet boundary condition for the computational-fluid-dynamics simulation.


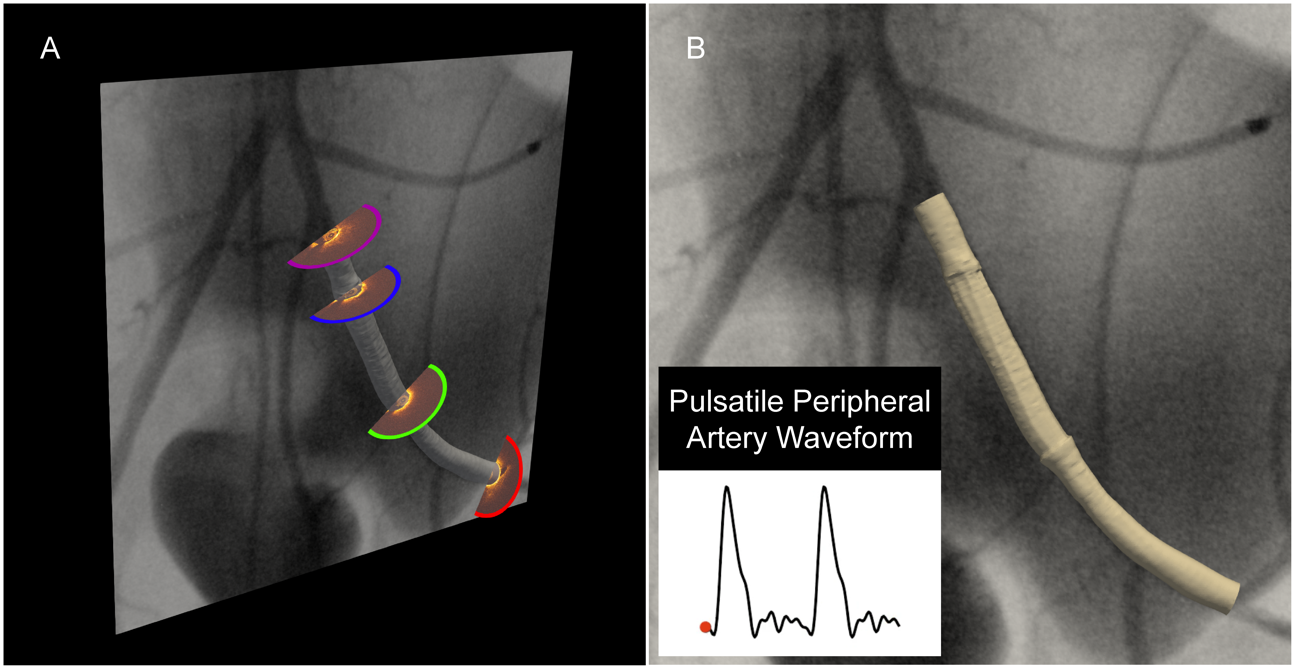


**Supplementary Figure 3.** Overview of final reconstructed vessel geometries used for CFD analysis. Three-dimensional luminal surface reconstructions for all vessels included in the computational fluid dynamics analyses are shown. Each panel displays a single reconstructed vessel geometry. Panel labels follow the format [Species-Animal number-Side-Time point], where Species denotes Rabbit or MiniPig; Animal number identifies the individual animal; Side denotes left (L) or right (R) vessel; and Time point denotes post-implantation baseline (PI), 1-month (1M), or 3-month (3M) follow-up imaging.


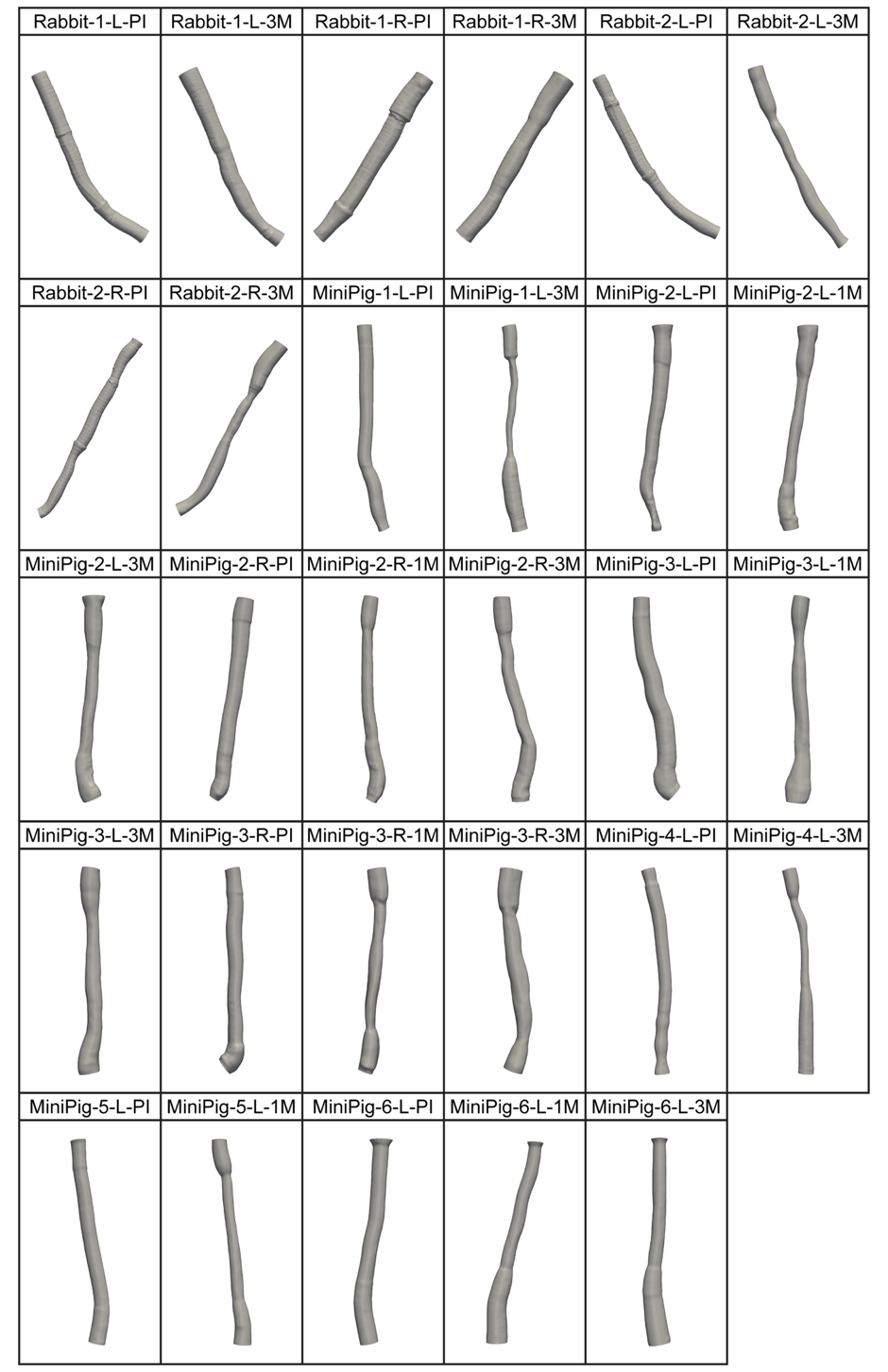


**Supplementary Figure 4.** Case-specific region of interest and scaffold length. **(A)** Absolute ROI length (mm) in mini-pigs (cases 1–6) and rabbits (cases 1–2) at post-implantation, 1 month, and 3 months, shown by vessel side (L, left; R, right). Solid (opaque) bars denote scaffold segments; semi-transparent bars denote peri-scaffold segments. **(B)** To account for subtle variations in OCT-derived length, despite meticulous serial co-registration, segment lengths were normalised to each ROI’s physical length, expressing scaffold position as a fraction of the unit-length (sum = 1).


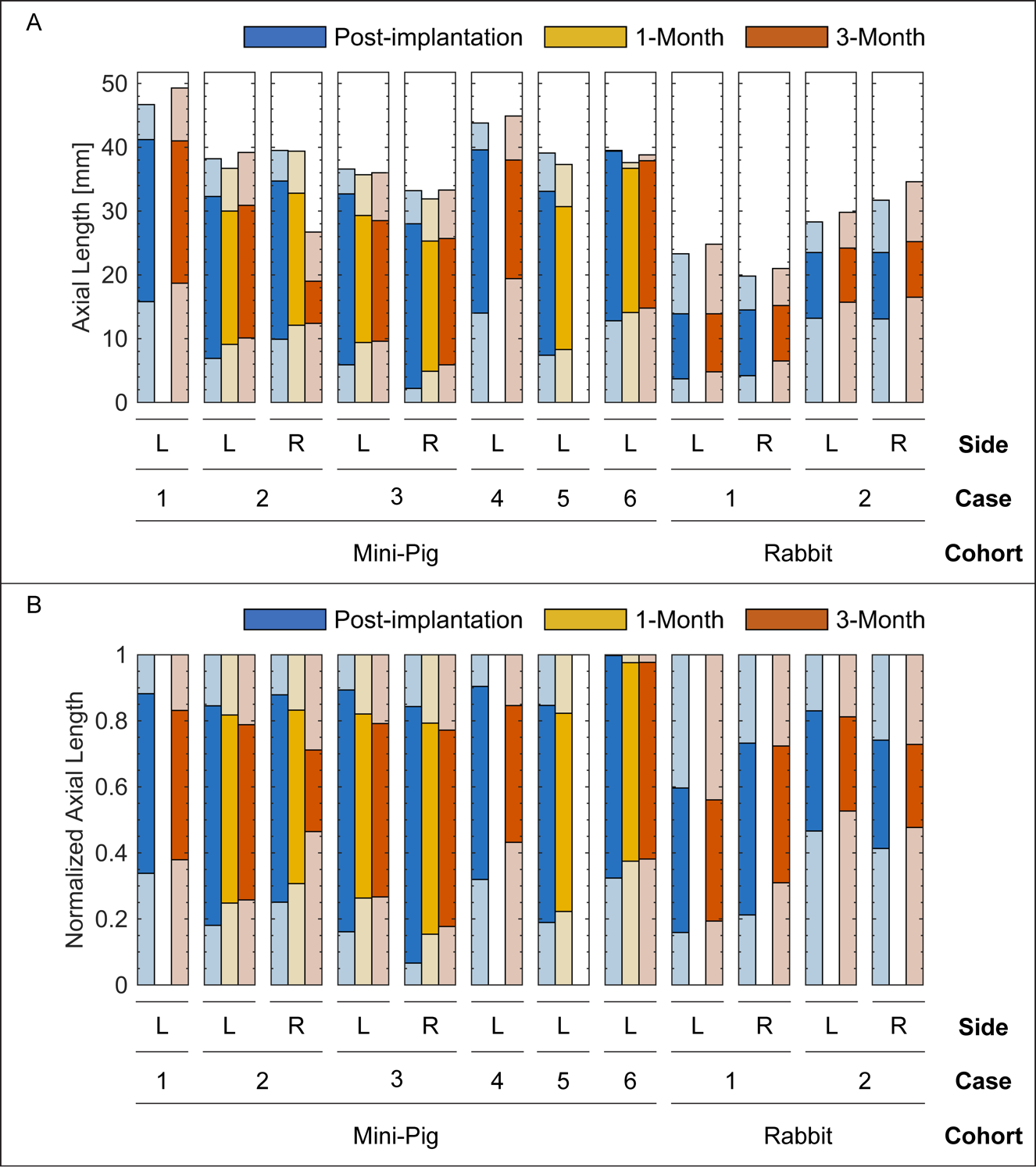


**Supplementary Figure 5.** Multidirectional endothelial shear stress in a representative rabbit iliac artery. Panels **(A)** show endothelial shear stress (ESS), **(B)** the spatial gradient of ESS (ESSG), **(C)** transverse ESS (transESS), **(D)** oscillatory shear index (OSI), and **(E)** relative residence time (RRT) at post-implantation. Corresponding maps at 3-month follow-up are shown in panels **A’-E’**. Data is mapped in ‘carpet view’ over normalized axial position (0-1) on the x-axis and circumferential length on the y-axis. The black horizontal bar above each map indicates the segment treated with the scaffold.


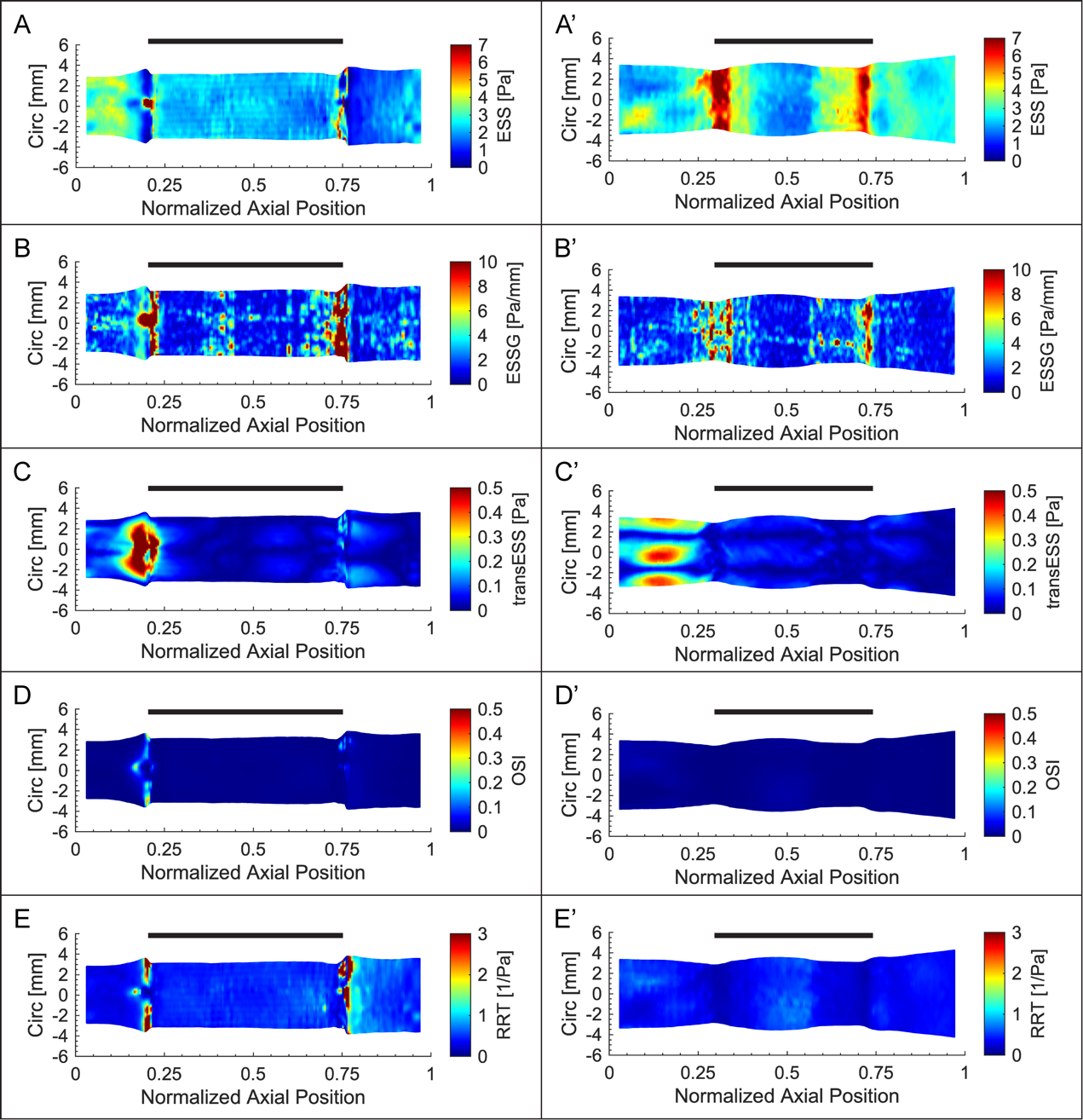


**Supplementary Figure 6.** Box‑and‑whisker plots of multidirectional shear metrics for all cases. **(A)** endothelial shear stress gradient (ESSG), **(B)** transverse endothelial shear stress (transESS), **(C)** oscillatory shear index (OSI), and **(D)** relative residence time (RRT) at post-implantation (blue), 1‑month (yellow) and 3‑month (red) follow-up for each case and vessel side (L, left; R, right). Boxes show the median and interquartile range (IQR); whiskers extend to the most extreme values within 1.5× IQR.


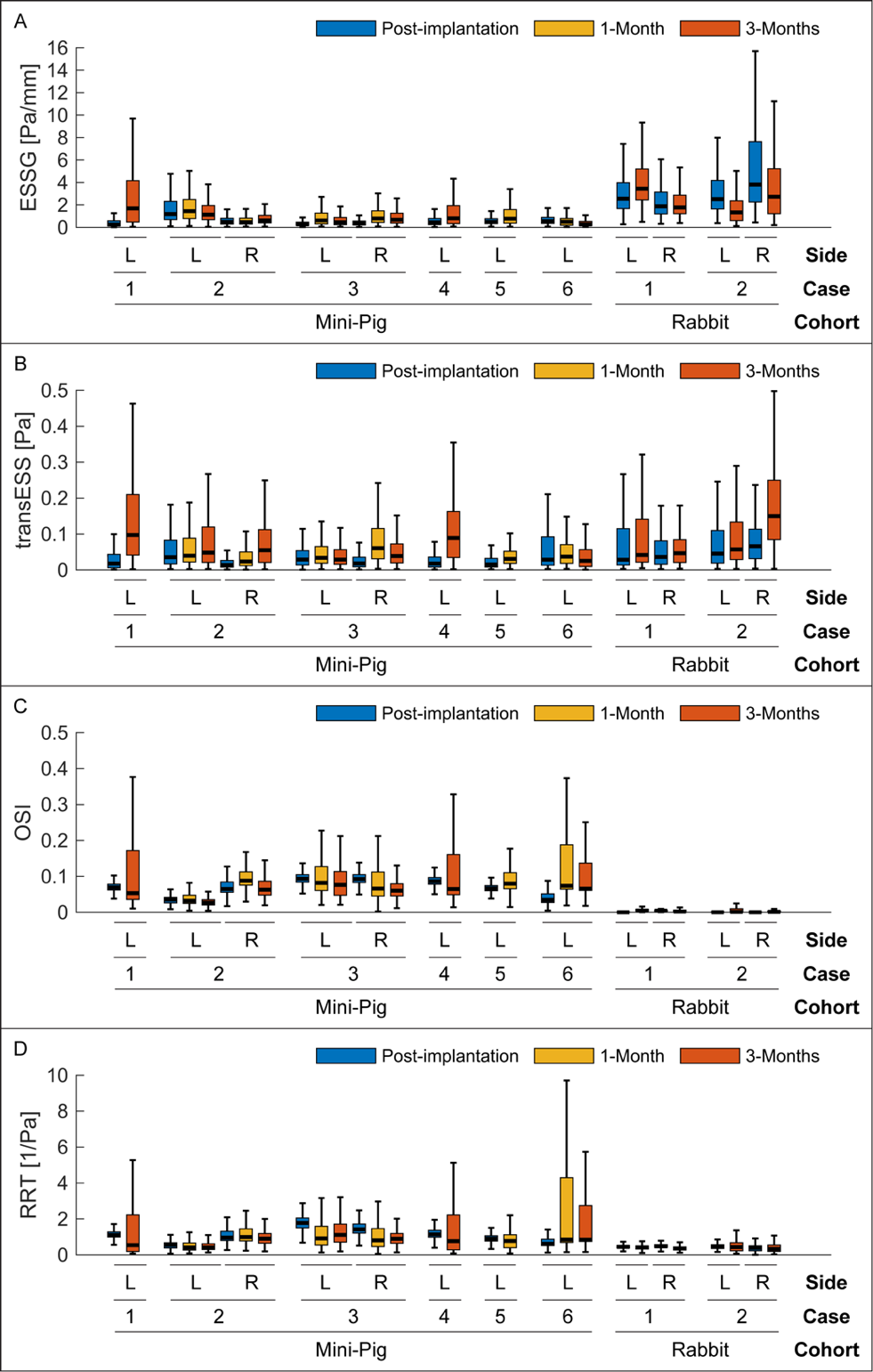
**Supplementary Video 1 (still image).** Intravascular OCT pullback in a rabbit immediately post-implantation, shown in axial and longitudinal views. The scaffolded lumen contour is annotated in blue, and red vertical lines mark the device’s entry and exit points. Within the scaffolded region, the device’s unique structure generates near-field scatter that shadows the underlying anatomy.

**
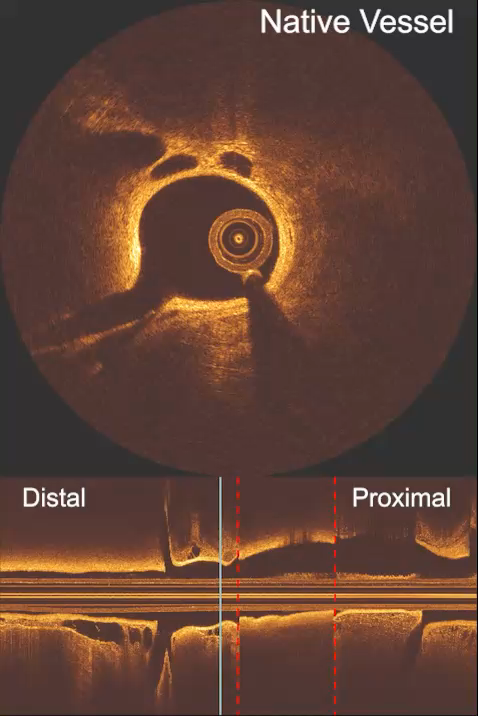
Supplementary Video 2 (still image).** Transient shear stress in a mini-pig 3D model at three time points (post-implantation, 1-month, and 3-month follow-up). The shear stress throughout the cardiac cycle is synchronized with the peripheral artery waveform displayed below each model.

**
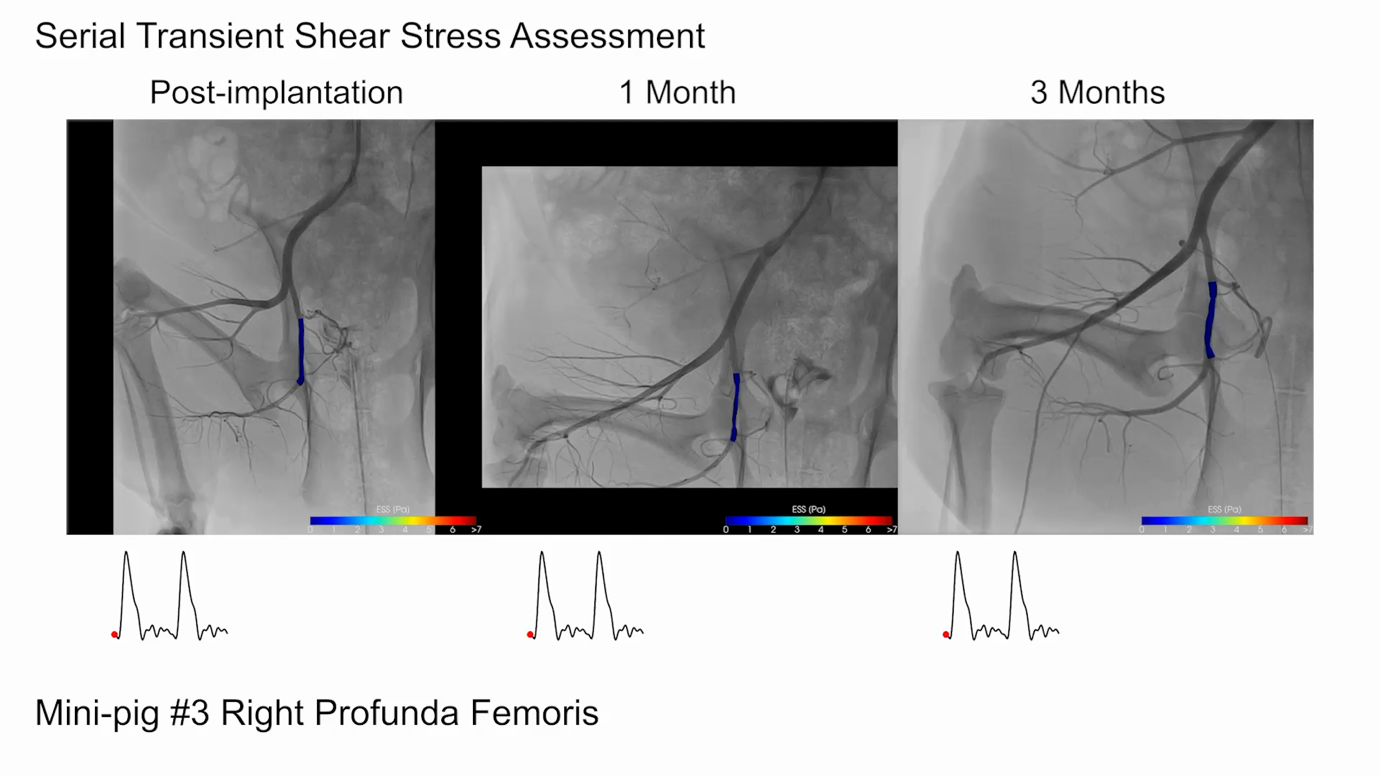
**
